# Decoding mechanisms of diarrhea induction by enteric viruses

**DOI:** 10.1371/journal.ppat.1012414

**Published:** 2024-08-08

**Authors:** Gaopeng Hou, Siyuan Ding

**Affiliations:** Department of Molecular Microbiology, Washington University School of Medicine in St. Louis, St. Louis, Missouri, United States of America; University of Iowa, UNITED STATES OF AMERICA

Infectious diarrhea kills more children each day than AIDS, malaria, and measles combined [1]. In humans, the leading causes of virally induced diarrhea include rotavirus, norovirus, sapovirus, astrovirus, adenovirus, and poliovirus, prior to a global vaccination program, all of which pose significant risks particularly to young children and the immunocompromised population [1]. Here, we summarize shared and distinct mechanisms employed by these viruses to cause diarrheal diseases. This brief review aims not only to serve and inform clinical and research communities but also paves the path for developing therapies and countermeasures to mitigate the impact of the diarrhea caused by these pathogens.

## Disruption of intestinal homeostasis

Rotavirus and norovirus are both capable of disrupting intestinal epithelial barriers but through different mechanisms. Rotavirus mainly infects mature non-proliferative enterocytes at the tip of villi in the small intestine and causes structural changes such as villus blunting in humans [2]. Breakdown of barrier function does not seem to be observed in mouse models of rotavirus infection, which could be due to increased stem cell division or neurotropic factor production [3,4]. This selective cell tropism towards mature absorptive enterocytes can lead to disruptions in normal intestinal physiology. Rotavirus infection also depresses the function of key apical transport proteins, such as sodium-D-glucose, sodium-hydrogen exchanger 3, and cystic fibrosis transmembrane conductance regulator, which are crucial for sodium and chloride transport in the small intestine [5]. Additionally, diacylglycerol O-acyltransferase 1 (DGAT1) is degraded in rotavirus antigen positive cells [5]. This degradation disrupts the localization of key ion transporters and reduces the expression of various intestinal proteins, leading to impaired nutrient absorption and malabsorptive noninflammatory diarrhea [5].

Human norovirus-induced diarrhea is associated with significant histopathological changes within the small intestine, despite minimal visible damage or inflammation. Key changes include the broadening and blunting of intestinal villi, vacuolization of epithelial cells, and hyperplasia of crypt cells [6]. These alterations, along with observed lacteal dilation, suggest impaired fat absorption and contribute to the malabsorptive aspect of diarrhea. Because human norovirus does not infect mice due to host range restriction, many of the disease studies stem from experimental murine noroviruses, whose cell tropism is strain dependent and largely found in subepithelial cells of the small intestinal gut-associated lymphoid tissue and lamina propria. Furthermore, the involvement of lymphocytes indicates a complex interplay where these cells help control viral replication but also exacerbate disease severity, highlighting a dual role in the pathogenesis of murine norovirus diarrhea [7]. Interestingly, murine norovirus infection only induces diarrhea in neonatal but not adult mice [7].

Murine astrovirus primarily infects goblet cells within the small intestine, a process that increases mucus production significantly [8]. This heightened mucus secretion likely disrupts normal intestinal function and alters the gut microbiome towards mucus-associated bacteria, potentially affecting the host susceptibility to other pathogens. Enhanced mucus production and altered gut microbiota can contribute to the malabsorption and disrupted electrolyte balance, leading to diarrhea. The infection driven increase in mucus can also modify the intestinal environment, affecting the overall homeostasis and immune responses, further contributing to diarrheal outcomes [8]. Human astrovirus infects different lineages of intestinal epithelial cells and infection induces significant alterations in the actin cytoskeleton and tight junction proteins, leading to increased cell permeability [9]. Astrovirus-infected cells display disrupted actin stress fibers and physical separation at the apical surface, indicating a breakdown in cell–cell adhesin. Moreover, occludin, a key component of tight junction, is redistributed to the cytoplasm, resulting in breaks in the cobblestone staining pattern characteristic of intact tight junctions [10]. These findings suggest a direct role of astrovirus in disrupting intestinal barrier integrity.

### Activation of the enteric nervous system

In addition to epithelial and immune cells, the enteric nervous system is involved in enteric virus-associated diarrhea development. Rotavirus infection primarily activates the secretion of serotonin from enterochromaffin cells, a subset of enteroendocrine cells [11]. This process is largely mediated by the viral nonstructural protein 4 (NSP4), which has been identified as a viroporin, which we will discuss below, and viral infection induced extracellular purinergic signaling [11]. The released serotonin acts on the serotonin receptors, which are key players in the gastrointestinal system, leading to increased intestinal motility and enhanced fluid secretion [11]. Studies have shown that blocking the serotonin receptors with small-molecule antagonists such as Ondansetron significantly reduces the severity and duration of diarrhea in infected mice, particularly in mouse pups more susceptible to rotavirus [11]. Moreover, serotonin receptors blockade also appears to reduce overall viral shedding and improves weight gain during infection, suggesting a direct link between serotonin signaling and viral replication [11]. The role of the enteric nervous system extends beyond just motility and fluid secretion; it also influences the viral impacts on electrolyte balance and intestinal inflammatory responses, further highlighting its critical role in the pathophysiology of rotavirus-induced diarrhea and presenting potential new avenues for therapeutic intervention. In a similar manner, enteric adenovirus, through its short fiber knob and hexon proteins, engages with enterochromaffin cells and stimulates serotonin release [12].

### Activities of viroporins

Viroporins are virally encoded proteins that oligomerize in the host membranes and form hydrophilic pores [13]. The mechanisms of diarrhea induced by the viroporin activities of rotavirus NSP4 and rhesus calicivirus Tulane virus NS1-2 highlight yet another shared viral strategy involving the manipulation of cellular calcium signaling to facilitate viral replication and induce gastrointestinal symptoms. As a viroporin localized to the endoplasmic reticulum (ER), NSP4 is the first viral enterotoxin discovered. It interacts with the stromal interaction molecule 1, depletes ER calcium stores, and triggers the activation of plasma membrane calcium influx channels [13]. Furthermore, rotavirus NSP4-induced calcium dysregulation is necessary for activation of purinergic intercellular calcium waves [14]. The resultant elevation in cytoplasmic calcium levels is crucial for viral replication and contributes to diarrhea by disturbing cellular functions and promoting secretory processes in intestinal cells. Similar to rotavirus NSP4, Tulane virus NS1-2 protein also functions as a viroporin within the ER, enhancing intracellular calcium signaling by facilitating the release of calcium from ER stores [15]. The NS1-2 protein specifically activates this process through its viroporin domain, which is essential for the effective replication of the viruses [15]. Increased cytoplasmic calcium, a direct outcome from NS1-2 activity, supports viral replication dynamics and is linked to the pathogenesis of diarrhea through disruption of cellular and systemic calcium balance. Three other examples of viroporins are Ebola virus delta peptide [16], astrovirus XP protein [17], and poliovirus 2B protein [18]. This highly convergent mechanism of diarrhea induction underscores the critical role of viroporins in viral life cycles and their impact on host gastrointestinal physiology. By inducing calcium imbalance, both NSP4 and NS1-2 not only ensure an environment conducive to viral replication but also contribute to the diarrheal output seen in infections, making these viroporin functions potential targets for therapeutic interventions aimed at mitigating disease symptoms and curtailing viral spread.

Importantly, SARS-CoV-2, a noncanonical enteric virus, also causes gastrointestinal symptoms. One potential mechanism is through an enterotoxin-like spike protein [19]. In a loop injection model, direct administration of SARS-CoV-2 spike protein induces intestinal lumen fluid accumulation, inflammation, mucosal histoarchitecture disruption, and altered innate immunity activation [19]. However, this has not been interrogated in the context of live virus infection nor the new variants and the underlying mechanisms are unclear.

### Induction of diarrhea by nonreplicating viral particles

There is one intriguing report that after psoralen UV treatment, transcription- and replication-defective rotaviruses can still cause diarrhea in suckling mouse pups [20]. This finding challenges the paradigm that diarrhea results solely from rotavirus replication-induced cell destruction and/or NSP4, which is only produced from cells with actively replicating viruses, highlighting a potentially alternative mechanism of viral attachment and entry into intestinal cells in diarrheal pathogenesis. Rotavirus-specific IgA response in the gut was not observed in mice receiving the oral inoculation of inactivated rotavirus [20], suggesting that if immunopathology is driving the diarrheal disease, it is more likely on the innate immune arm of the host response. These findings open new avenues for understanding the pathogenesis of rotavirus-induced diarrhea and suggest potential targets for therapeutic intervention.

## Conclusions

The multifaceted mechanisms by which enteric viruses induce diarrhea emphasize the complex interaction between the enteric viruses and the host gastrointestinal system. From the disruption of intestinal barriers to the activation of the enteric nervous system, the pivotal role of viroporins and the nonreplicating viruses (**Table 1**), these findings illuminate novel avenues for prophylactic and therapeutic interventions. Thus, continued exploration of mechanisms of viral pathogenesis holds promise for the development of broad-spectrum antivirals and vaccines, with the goal of reducing overall infection-associated diarrheal illness and sequelae.

**Table 1:** Mechanisms of diarrhea induction by viruses.

Causes of diarrhea	Enteric viruses	Cellular mechanisms
Gut homeostasis disruption	Rotavirus, norovirus, and astrovirus	Intestinal villus blunting, apical transporter protein mis-localization, and tight junction protein alteration
Enteric nervous system activation	Rotavirus, adenovirus, and SARS-CoV-2	Intestinal serotonin secretion and altered gut motility
Viroporin function	Rotavirus, rhesus calicivirus, astrovirus, Ebola virus, poliovirus, and SARS-CoV-2?	Cellular calcium signaling dysregulation and beyond
Host immune response?	Non-replicating rotavirus	Unknown
